# Progress in Childhood Vaccination Data in Immunization Information Systems — United States, 2013–2016

**DOI:** 10.15585/mmwr.mm6643a4

**Published:** 2017-11-03

**Authors:** Neil Murthy, Loren Rodgers, Laura Pabst, Amy Parker Fiebelkorn, Terence Ng

**Affiliations:** ^1^Epidemic Intelligence Service, CDC; ^2^Immunization Services Division, National Center for Immunization and Respiratory Diseases, CDC.

In 2016, 55 jurisdictions in 49 states and six cities in the United States* used immunization information systems (IISs) to collect and manage immunization data and support vaccination providers and immunization programs. To monitor progress toward achieving IIS program goals, CDC surveys jurisdictions through an annual self-administered IIS Annual Report (IISAR). Data from the 2013–2016 IISARs were analyzed to assess progress made in four priority areas: 1) data completeness, 2) bidirectional exchange of data with electronic health record systems, 3) clinical decision support for immunizations, and 4) ability to generate childhood vaccination coverage estimates. IIS participation among children aged 4 months through 5 years increased from 90% in 2013 to 94% in 2016, and 33 jurisdictions reported ≥95% of children aged 4 months through 5 years participating in their IIS in 2016. Bidirectional messaging capacity in IISs increased from 25 jurisdictions in 2013 to 37 in 2016. In 2016, nearly all jurisdictions (52 of 55) could provide automated provider-level coverage reports, and 32 jurisdictions reported that their IISs could send vaccine forecasts to providers via Health Level 7 (HL7) messaging, up from 17 in 2013. Incremental progress was made in each area since 2013, but continued effort is needed to implement these critical functionalities among all IISs. Success in these priority areas, as defined by the IIS Functional Standards (*1*), bolsters clinicians’ and public health practitioners’ ability to attain high vaccination coverage in pediatric populations, and prepares IISs to develop more advanced functionalities to support state/local immunization services. Success in these priority areas also supports the achievement of federal immunization objectives, including the use of IISs as supplemental sampling frames for vaccination coverage surveys like the National Immunization Survey (NIS)-Child, reducing data collection costs, and supporting increased precision of state-level estimates.

IISs, also known as immunization registries, are confidential, computerized, population-based systems that collect and consolidate vaccination data from providers in a jurisdiction (*2*). IISs increase vaccination rates and reduce vaccine-preventable diseases by enabling effective interventions (e.g., client reminder and recall, provider assessment and feedback), tracking patient immunizations, estimating vaccination coverage, and facilitating vaccine management and accountability (*3*). For IISs to support real-time immunization efforts both at the population level and at the point of clinical care, these systems need to capture complete childhood immunization data. To promote IIS functionality and data quality, CDC and external partners, including state/local immunization programs and IIS vendors, developed 27 Functional Standards to guide IIS development from 2013 to 2017 (*1*). CDC monitors progress toward these Functional Standards through a self-administered survey known as the IIS Annual Report (IISAR). During 2016–2017, CDC issued guidance to jurisdictions identifying four priority areas (covering multiple Functional Standards) that immunization programs should focus on before developing other IIS functionalities. The four priority areas are: 1) data completeness for children aged 0–6 years (Functional Standard 1.1, 3.1); 2) bidirectional information exchange with electronic health record systems (1.4, 1.5); 3) pediatric clinical decision support for immunizations (1.2), and 4) ability to generate jurisdictional and provider-level childhood vaccination coverage estimates (5.2). This report assesses progress toward achieving success in these four priority areas from 2013 to 2016, using data from the 2013–2016 IISARs. IISAR is a secure web-based survey instrument distributed annually to state, local, and territorial immunization programs by CDC. Immunization programs self-report their IIS’s progress toward meeting the Functional Standards during the previous calendar year.

Data completeness comprises four measures: birth record capture, child participation, provider participation, and IIS coverage estimate comparison to NIS-Child. These measures represent the ability of an IIS to capture the population within the jurisdiction as well as all vaccinations administered. Birth record capture is defined as the ability of an IIS to create patient records for all children who are born in a jurisdiction. Child participation is defined as the number of children aged 4 months through 5 years with ≥2 vaccinations recorded in the IIS, divided by the total U.S. Census–based population estimate for the same age group in that jurisdiction. Provider participation is defined as the number of vaccination provider sites enrolled in an IIS that reported ≥1 vaccine doses to the IIS within the last 6 months of the preceding calendar year. IIS participation among the >40,000 provider sites served by the publicly funded Vaccines for Children (VFC) program^†^ was analyzed. The comparison of IIS coverage estimates with estimates from NIS-Child measures an IIS’s success in capturing complete population and vaccination information within a jurisdiction.^§^

Across all IIS jurisdictions, 106%^¶^ of U.S. births were captured in IIS in 2016, an increase from 102% in 2013. Childhood IIS participation increased from 90% in 2013 to 94% in 2016, which approaches the *Healthy People 2020* objective of ≥95% child IIS participation. Among the 55 jurisdictions, 33 (60%) reported that ≥95% of children aged 4 months through 5 years in their geographic area participated in their IIS in 2016, compared with 24 (44%) in 2013. In 2016, provider participation was 85% among VFC provider sites enrolled in an IIS. The number of VFC provider sites enrolled in an IIS decreased from 41,710 in 2014 to 41,393 in 2016. Among these enrolled sites, the number of VFC provider sites participating in an IIS increased slightly from 33,266 in 2013 to 34,662 in 2016 (Figure 1).

**FIGURE 1:**
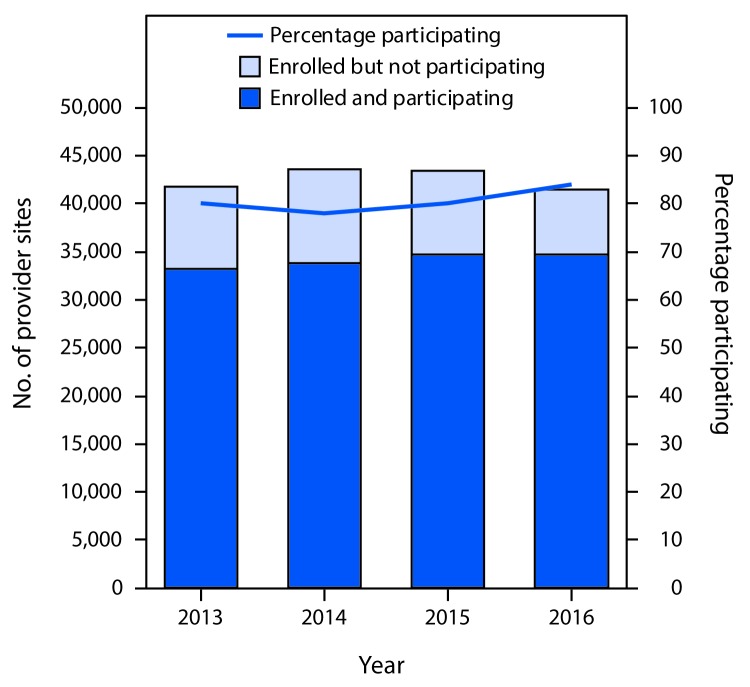
Number and percentage of Vaccines for Children program provider sites enrolled and participating* in an Immunization Information System (IIS), by year — IIS Annual Report, United States, 2013–2016 * Participation is defined as having submitted information to the IIS about administering ≥1 vaccine dose in the last 6 months of the preceding calendar year. Provider sites must be enrolled in an IIS to participate in the IIS.

For the combined 7-vaccine series,** the number of jurisdictions with IIS estimates within 10 percentage points of the corresponding NIS-Child coverage estimates increased from 17 in 2013 to 25 in 2016 (Figure 2). In 2016, 30 IISs had 7-vaccine series coverage estimates that were at least 10 percentage points lower than the corresponding NIS-Child estimate.

**FIGURE 2:**
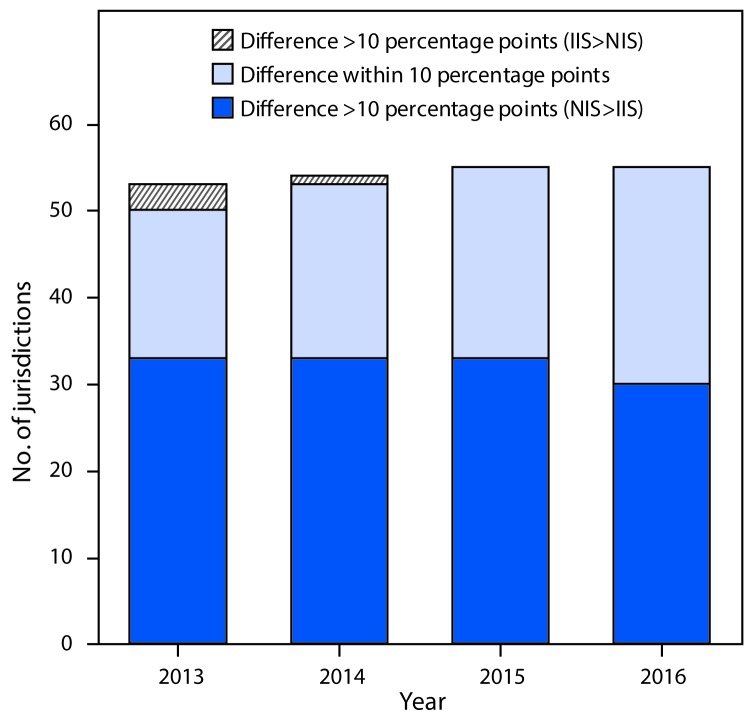
Percentage point differences between National Immunization Survey (NIS)-Child and Immunization Information Systems (IISs) for combined 7-vaccine series* completion — IIS Annual Report, United States, 2013–2016 * ≥4 doses of diphtheria and tetanus toxoids and acellular pertussis vaccine; ≥3 doses of poliovirus vaccine; ≥1 doses of measles-containing vaccine; *Haemophilus influenzae* type B vaccine full series; ≥3 doses of hepatitis B vaccine; ≥1 dose of varicella vaccine; and ≥4 doses of pneumococcal conjugate vaccine.

Bidirectional information exchange allows providers to submit immunization data directly from electronic health records (EHRs) to IISs, and to request and receive immunization information from IISs into EHRs for the patients they serve. HL7 messaging is a nationally recognized platform-independent standard that supports the bidirectional exchange of health-related information, including immunization-related messaging. In 2016, 91% of jurisdictions had an IIS that used HL7 version 2.5.1 to receive vaccination histories from providers and returned acknowledgment messages, compared with 87% in 2013. Furthermore, in 2016, 67% of jurisdictions had an IIS that received requests for vaccination histories and returned responses to those requests, compared with 45% in 2013 (Figure 3). Finally, in 2016, 78% of jurisdictions had an IIS that could transmit immunization data using Simple Object Access Protocol, the CDC-endorsed transport standard for the exchange of immunization information, compared with 75% of jurisdictions reporting this capability in 2013 (*4*).

**FIGURE 3:**
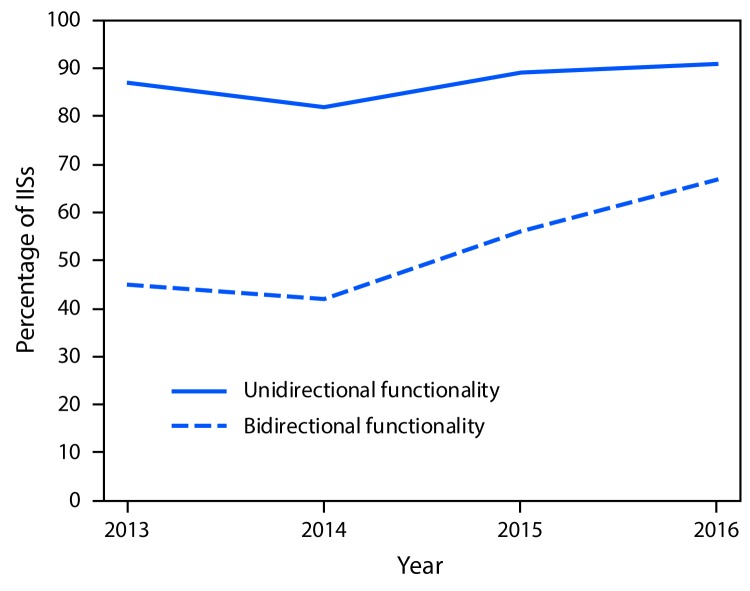
Percentage of Immunization Information Systems (IISs) with unidirectional and bidirectional information exchange functionality* — United States, 2013–2016 * Unidirectional functionality is defined as the ability to receive vaccination histories (message type: VXU) from providers and return acknowledgment messages (message type: ACK), and bidirectional functionality is defined as the ability to receive requests for vaccination histories (message type: QBP) and return responses to those requests (message type: RSP). Achievement of unidirectional functionality is a prerequisite to achieving bidirectional functionality. https://www.cdc.gov/vaccines/programs/iis/technical-guidance/downloads/hl7guide-1-5-2014-11.pdf.

Clinical Decision Support (CDS) functionalities enable providers to evaluate the validity of vaccine doses administered to patients and forecast future vaccines that will be needed, based on recommendations developed by the Advisory Committee on Immunization Practices. From 2013 to 2016, all jurisdictions’ IISs had CDS capabilities that were available to providers through the IIS’s user interface. In 2016, 58% (32 of 55) of jurisdictions reported sending a vaccine forecast to another system via HL7 messaging. This is an 87% increase from 2013, when 31% (17 of 55) of jurisdictions reported performing this task.

IISs can be used to generate coverage estimates for childhood vaccinations at the jurisdictional level (e.g., state, postal code, or county) and at the provider level to identify vulnerable subpopulations. In 2016, 89% of jurisdictions (49 of 55) provided a predefined, automatic report on immunization coverage by geography. This is 11% higher than in 2013, when 80% of jurisdictions provided these reports. In 2016, 95% of jurisdictions (52 of 55) provided a predefined, automatic report on immunization coverage by provider site. This is 7% higher than in 2013, when 89% of jurisdictions reported providing these reports.

## Discussion

Since 2013, incremental progress was noted in each of the four priority areas for immunization programs that were assessed. Notably, the increased number of jurisdictions that had IIS estimates that were within 10 percentage points of the corresponding NIS-Child coverage estimate suggests that more jurisdictions have IISs with more complete data, or at least that the IIS and NIS are similar in their ability to estimate vaccination coverage for that jurisdiction’s population. Jurisdictions with IIS coverage estimates that were at least 10 percentage points lower than the corresponding NIS-Child estimate might have less complete IIS data, particularly at sites with the largest IIS–NIS discrepancies.

By prioritizing resources to the identified priority areas, jurisdictions can make substantial progress in this important subset of activities rather than incremental progress across all Functional Standards. Improvements in priority areas can also support a broader range of immunization services; for example, improved data completeness for children aged <6 years would strengthen immunization delivery for this population (Functional Standard 1.1–1.3) and increase VFC program accountability (2.1–2.6). In addition, as IISs identify more children and record all doses administered within their jurisdiction, IIS-based vaccination coverage estimates will be able to supplement estimates from surveys like the NIS-Child (*5*). IISs are integral components of routine clinical practice and public health surveillance for immunization. Availability of more complete IIS data also offer many benefits to health care providers and public health practitioners, including consolidating patients’ vaccination histories, identifying undervaccinated subgroups, and forecasting the needs of individual patients for recommended vaccines (*3*).

Standards and best practices exist that can guide IIS development and maintenance activities, including the IIS Functional Standards (*1*), national standards for the electronic exchange of immunization information,^††^ CDS resources,^§§^ and data quality best practices.^¶¶^Alignment with these standards and best practices reduces variability across IISs and helps IISs use resources more efficiently to provide the most value for immunization programs, providers, patients, and parents. Continuously monitoring the progress of each IIS can also help jurisdictions identify areas for improvement. Such monitoring is done using the IISAR or other tools, such as an initiative to assess, measure, and validate IISs that was recently developed by the American Immunization Registry Association (*6*).

The findings in this report are subject to at least three limitations. First, results were self-reported and might be subject to response bias. Second, only a subset of the Functional Standards pertaining to the four priority areas was analyzed in this report; this evaluation was not a comprehensive analysis of the progress made in all Functional Standards. Finally, reported capacity of a functionality does not necessarily indicate active utilization of that functionality.

This was the first systematic assessment of progress in four priority areas that are foundational for IISs. Incorporating strategies such as prioritizing activities, aligning resources, implementing best practices, adhering to national standards, and implementing independent third-party assessments can promote consistency across jurisdictions, encourage program accountability, ensure quality standards, and help IISs more rapidly attain their full potential to facilitate complete vaccination of U.S. children against vaccine-preventable diseases.

**Summary**

**What is already known about this topic?**

In 2012, 86% of U.S. children aged 4 months through 5 years (19.5 million) had ≥2 doses recorded in immunization information systems (IISs).

**What is added by this report?**

From 2013 to 2016, the percentage of children with ≥2 immunizations recorded in IISs increased from 90% to 94%, approaching the *Healthy People 2020* objective of ≥95%. However, variability in IIS pediatric data quality persists: 30 of 55 IISs produced 7-vaccine series coverage rates that were at least 10 percentage points lower than the corresponding National Immunization Survey-Child coverage rate in 2016, suggesting incompleteness of IIS data. Across all IISs, there was progress in achieving bidirectional information exchange with electronic health record systems, pediatric clinical decision support for immunizations, and the ability to generate jurisdictional and provider-level childhood vaccination coverage estimates.

**What are the implications for public health practice?**

To realize the full benefits of IISs, immunization programs need to implement strategies that prioritize and align resources to achieve functionality and high data quality in four focus areas: 1) pediatric data completeness, 2) bidirectional data exchange with electronic health record systems, 3) clinical decision support for immunizations, and 4) ability to generate childhood vaccination coverage estimates. Strategies such as implementing best practices, adhering to national standards, and incorporating independent third-party assessments can reduce variability across IISs, and support IIS’ full potential to facilitate complete vaccination of U.S. children against vaccine-preventable diseases.

## Footnotes

* Excluding the U.S. territories.

† The Vaccines for Children program provides vaccines at no cost to eligible children, including those whose parents or guardians might not be able to afford vaccines. https://www.cdc.gov/vaccines/programs/vfc/index.html.

§ NIS-Child is a nationally representative survey that estimates vaccination coverage among children aged 19**–**35 months using provider-reported vaccination data. Many factors can potentially affect the calculated percentage point differences between IIS and NIS-Child estimates, including possible errors in IIS vaccination and population calculations, errors in NIS-Child (such as random error, selection bias, and underascertainment of vaccination status), variability in data completeness in individual IIS, and other methodological differences between NIS-Child and IISs. Despite these limitations, the NIS-Child is well established as a reliable indicator of childhood vaccination rates, and serves as a useful holistic benchmark for assessing an IIS’ performance in capturing recorded doses administered within its jurisdiction.

¶ Comparisons were made with U.S. Census estimates for children aged <1 year in 2016. Birth record capture percentages often exceed 100% because of newborn data being recorded in more than one IIS (e.g., a child who is born in 1 state but who is a resident in a different state might be recorded in both IISs), and because of incomplete inactivation of records of children who move out of a jurisdiction.

** ≥4 doses of diphtheria and tetanus toxoids and acellular pertussis vaccine; ≥3 doses of poliovirus vaccine; ≥1 dose of measles-containing vaccine; *Haemophilus influenzae* type B vaccine full series; ≥3 doses of Hepatitis B vaccine; ≥1 doses of varicella vaccine; and ≥4 doses of pneumococcal conjugate vaccine.

†† IIS HL7 Implementation: https://www.cdc.gov/vaccines/programs/iis/technical-guidance/hl7.html; IIS Transport (SOAP): https://www.cdc.gov/vaccines/programs/iis/technical-guidance/soap/services.html.

§§ IIS Clinical Decision Support for Immunization (CDSi): https://www.cdc.gov/vaccines/programs/iis/cdsi.html.

¶¶ American Immunization Registry Association: Data Quality Assurance in IIS: http://www.aira.browsermedia.com/resources/AIRA-MIROW_DQA_Selected_Aspects_best_practice_guide_05-17-2013.pdf; American Immunization Registry Association: Data Visualization Guide: http://repository.immregistries.org/files/resources/58a601d626d7a/aira_data_validation_guide.pdf; IIS Deduplication: https://www.cdc.gov/vaccines/programs/iis/technical-guidance/deduplication.html.
